# Unsupervised Moving Object Segmentation from Stationary or Moving Camera Based on Multi-frame Homography Constraints

**DOI:** 10.3390/s19194344

**Published:** 2019-10-08

**Authors:** Zhigao Cui, Ke Jiang, Tao Wang

**Affiliations:** Xi’an Research Institute of High-Tech, Xi’an 710025, China; Jiangke1019@163.com (K.J.); Wangtao09270927@163.com (T.W.)

**Keywords:** moving object segmentation, motion trajectory, multi-frame homography constraint, Markov random fields model

## Abstract

Moving object segmentation is the most fundamental task for many vision-based applications. In the past decade, it has been performed on the stationary camera, or moving camera, respectively. In this paper, we show that the moving object segmentation can be addressed in a unified framework for both type of cameras. The proposed method consists of two stages: (1) In the first stage, a novel multi-frame homography model is generated to describe the background motion. Then, the inliers and outliers of that model are classified as background trajectories and moving object trajectories by the designed cumulative acknowledgment strategy. (2) In the second stage, a super-pixel-based Markov Random Fields model is used to refine the spatial accuracy of initial segmentation and obtain final pixel level labeling, which has integrated trajectory classification information, a dynamic appearance model, and spatial temporal cues. The proposed method overcomes the limitations of existing object segmentation algorithms and resolves the difference between stationary and moving cameras. The algorithm is tested on several challenging open datasets. Experiments show that the proposed method presents significant performance improvement over state-of-the-art techniques quantitatively and qualitatively.

## 1. Introduction

Unsupervised moving object segmentation is a challenging problem for many applications, such as video semantic analysis, intelligent transportation system, automated video surveillance [1], and so on. In the unsupervised manner, the algorithm should segment the foreground moving objects from complex videos automatically, where cluttered backgrounds [2,3], scale diversification, and motion blurs exist. In a previous study, various unsupervised algorithms have been proposed to deal with the videos captured by the stationary camera, where the camera does not move and the scene in the video does not change. Generally speaking, a popular method is in generating a background model of the scene representation, and then the outliers of that model are treated as moving objects. In the past decades, a significant number of literatures have been published under the assumption that the camera is stationary, such as Gaussian Mixture Model used in [4], Codebook Model used in [5], and Self-Organizing Neural Network Model proposed by Maddalena and Petrosino [6]. However, the captured videos may not be static in many real applications. For example, pan-tilt-zoom (PTZ) cameras [7,8] have been widely used in modern surveillance systems recently, whose view can be dynamically controlled by their panning, tilting, and zooming parameters. In addition, the videos used for sematic analysis are almost captured by the handheld cameras. In such cases, the background subtraction methods used in the stationary background circumvent cannot be applied directly. Therefore, a moving segmentation algorithm, that can solve freely moving cameras, is necessary. And a unified segmentation framework, for the both types of cameras, also needs to be present.

### 1.1. Related Work

Previous methods for moving object segmentation are vast, especially for the stationary camera. While, our method aims to introduce a segmentation technique for the moving camera, which can be extended to the stationary camera case automatically. Thus, motion segmentation for the moving camera is the most related topic to this paper. When the camera is moving, the pixels corresponding to background do not maintain the same image positions in consecutive frames, which severely make the traditional video object segmentation complicated. In order to segment the object regions, an intuitive idea is to estimate the transformation parameters between consecutive images and creating a difference image [9,10,11]. By now, most of the printed literatures are aware this approach, and this it has become widely adopted as it requires less computational cost and memory storage. However, this approach only returns an incomplete object contour. Another body of work has attempted to extend stationary background modeling methods for moving cameras. Then, moving objects can be segmented in a similar way to the fixed camera case. Xue et al. [12] introduced a panoramic Gaussian Mixture Model, which provides global information for the moving camera’s field of view. Then, each observed frame is registered to the panoramic background to segment moving objects. However, there are many limitations of panorama-based methods, such as error accumulation, slow background adaptation, and so on. Instead of constructing a large panoramic background model, Kim et al. [13] build a spatio-temporal background model, whose size is same as observed frame. Then, Lucas Kanada Tracking method [14] is adopted to estimate camera motion between observed frame and background model, and the background subtraction technology is used to segment moving objects. The limitation of this method is that the moving camera must keep static at first to generate key frames and initialize the background model. Using similar strategy, Ferone and Maddalena [15] proposed an extension method of their self-organizing neural network model [6] to the case of moving cameras. However, as they stated in the experiments, a hand-made initial background estimate is needed if the video sequences have no initial static frames for background estimation.

Indeed, both of the above methods use motion and color information between consecutive images to distinguish between moving objects and background. While, the motion cues [16] accumulated in the multiple frames are not utilized, the researchers attempted to extract a long term trajectory [17], which can establish point correspondence between multiple frame images. With these point trajectories, the differences between moving object trajectories and background trajectories can be measured and the classification can be implemented. Dey et al. [18] constructs a monocular multi-frame epipolar constraint, then point trajectories that violate the constraint are regarded as moving objects. However, their method just segment moving objects from the background at the trajectory level, and the final pixel level classification is not considered. Thus, the segmentation results tend to be sparse and incomplete. Ochs and Brox [19] obtain dense segmentation results by combining spectral clustering method and a mulit-level variational model. Their method, however, incorporates little dynamic appearance and spatial temporal information for the final labeling, which may show bad performance in complicated and challenging videos. On the other hand, [20] proposes a matrix factorization method, based on low rank [21] and group sparsity constraints. Their method is sensitive to incomplete trajectories, especially when the camera is moving fast. In [22], the authors assume that the background trajectory is located on the low rank subspace, composed of three basic trajectories [23] under the assumptions of affine camera model, whereas the foreground trajectory deviates from the subspace. However, this method is hindered by the following limitations. First, an affine camera model, rather than a more accurate perspective camera model, is used, thereby resulting in poor segmentation performance for considerable changes in background depth. Second, this method needs the long term trajectory to be equal length, which will make part of the image region have no trajectory point. Third, fixed rank constraints face difficulty in managing a stationary camera circumvent (at this point, the rank of the observation matrix for the background trajectory is two [24,25]). Recently, Zhu et al. [26] proposes a multilayer based framework for online background subtraction for videos captured by moving camera. While, their method is only adapted to the moving camera only, their method is not stable and sensitive to parameter selection.

Besides long term trajectory, the frame-to-frame dense optical flow also contains rich motion information. Using optical flow, Lim et al. [27] proposes an iterative method by dividing the image into multiple blocks and estimating the background and foreground motion of each block. However, their method is prone to complex scene and small objects that inhibit the planar scene assumption for each block. In [28], the authors use a similar strategy, which is based on density propagation techniques of image block, but their methods are complex and have many free parameters involved. The works presented in [29,30] attempt to discover key-segments and group them to find the foreground objects by measuring multi-scale appearance and optical flow. While, their method does not deal with the moving object segmentation problem, it just output a ranked list of spatio temporal segments likely to be objects. On the other hand, authors in [31] proposes to segment the prominent moving objects based on iterative refinement with neighborhood information. Their method relies too much on the results of bottom-up saliency detection approaches [32], which may not give the desired result since they are very prone to errors by now. Yang et al. [33] proposes an adversarial contextual model to segment moving object from the camera. Their method uses the deep neural network to predict the optical flow in a region and works on a supervised manner.

### 1.2. Our Contributions

In this paper, we present a unified framework for moving object segmentation from a moving camera or stationary camera, based on a multi-frame homography constraints. Unlike [20,22], we do not apply a low-rank model. Instead, we divided the whole video into several overlapped windows, and generate a multi-frame homography model for the background. This treatment can utilize all the trajectories in the video, and overcome the aforementioned problems of [20,22]. On the other hand, unlike [27], we do not assume that the scene is planar or the camera motion is in rotation. The only requirement is that the object is moving differently from its surrounding background. Therefore, the proposed method can handle rapidly moving background, arbitrary object motion, non-rigid deformations, and so on.

**Figure 1:**
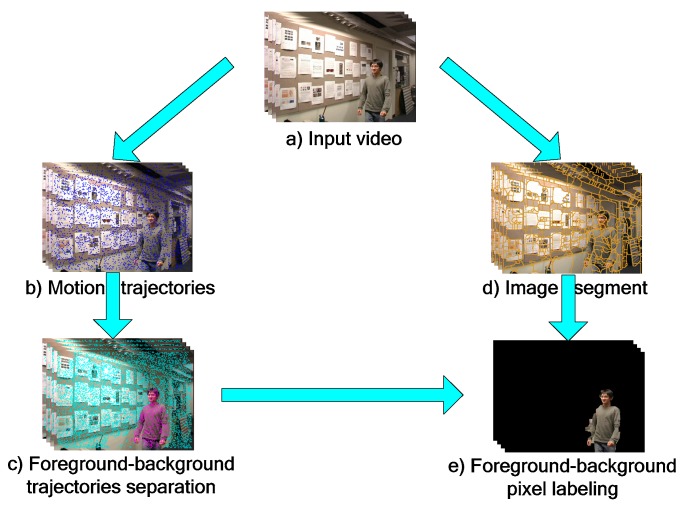
The framework. Our method takes a raw video as input, and produces a binary labeling as the output. Two major steps are initial multi-frame homography model for trajectories classification (left) and final Markov random fields model for foreground background labeling (right).

Figure 1 shows the graphical abstract of the proposed scheme. The proposed segmentation algorithm takes a raw video sequence as input, and generates a binary labeling at the pixel level. It has two major steps: Initial multi-frame homography model for trajectories classification and final Markov Random Fields model for foreground background labeling. In the first step, a dense set of trajectories are tracked over all frames. With the dense point trajectories, a novel multi-frame homography model is proposed to describe the background motion. Then, applying the designed cumulative acknowledgment method, the inliers and outliers of that model are treated as background trajectories, and moving object trajectories, respectively. In the second step, a superpixel-based Markov Random Fields model is built to label motion segments as foreground or background, which has incorporated spatial temporal smoothness of each superpixel and dynamic appearance of moving object and background.

The contributions of this paper can be summarized as follows. We introduce a unified framework for automatic video object segmentation from moving camera or stationary camera by: (1) constructing a multi-frame homography model that relates adjacent frames in the whole video; (2) designing a trajectory classification method based on cumulative acknowledgment strategy; (3) incorporating trajectory classification, dynamic appearance, spatial temporal cues for the final labeling.

The remainder of the paper is organized as follows. In the next section, we introduce the multi-frame homography model and cumulative acknowledgment strategy for trajectories classification. Then, the details of proposed foreground-background labeling method are presented in Section 3. Section 4 provides the experimental results and comparative study with recent state-of-the-art techniques. Finally, Section 5 concludes this paper.

## 2. Trajectory Classification Based on Multi-Frame Homography Model

We use an off-the-shelf dense point tracker [34] to produce the trajectories. Compared to traditional feature point trackers, this method can provide arbitrarily dense trajectories, so it allows labels to be assigned more densely. We assume that the calculated trajectories are expressed as,

(1) $$\Lambda =\left\{\Lambda_{i},i=1,\ldots ,n\right\},\Lambda_{i}=\left\{x_{i}^{l},l=t_{i},\ldots ,T_{i}\right\}$$

where n is the total number of produced trajectories; $t_{i}$ and $T_{i}$ are the initial and end frame numbers, respectively, of the trajectory $\Lambda_{i}$; and $x_{i}^{l}$ represents the homogeneous coordinates of trajectory $\Lambda_{i}$ on the frame $l\left(t_{i}\leq l\leq T\right)$. In general, the produced trajectories can be divided into two categories: the background trajectories generated by the motion of the camera, and the foreground trajectories generated by the moving object. Given the tracked trajectories, our objective is to estimate the foreground support as well as the underlying background ones.

### 2.1. Homography Constraint

In view of the smooth camera movement in a typical video sequence, the change of camera center in the adjacent frames is small, so that the background motion can be approximated by the homography constraint [35,36].

(2) $$x^{\prime }\approx Hx$$

The aforementioned constraints can be understood as follows. For a video sequence acquired by a handheld camera, the background of the adjacent frames can approximately satisfy the homography constraints. For a video sequence acquired by a PTZ camera, since the optical center is assumed to remain unchanged, so the background points of adjacent frames strictly satisfy the homography constraints. While, for a video sequence acquired by a stationary camera, the homography model is degraded to the identity matrix. Thus, the homography model can be used to establish the background motion constraint of adjacent frames for a plurality of video sequences (stationary camera or moving camera).

### 2.2. Trajectory Classification Method

With the above homography constraint, we propose a novel trajectory classification method. Firstly, we divided the input video temporally into T-t overlapping windows of T frames with the time interval t. Subsequently, the algorithm estimates the homography constraint of each window separately. Thus, we could obtain the homography matrix set of all windows, which is a rich description of the background motion. Finally, to obtain an accurate classification of tracked trajectories, we propose a mechanism to combine the results of a consecutive homography matrix to analyze the accumulative motion properties of each trajectory.

**Figure 2:**
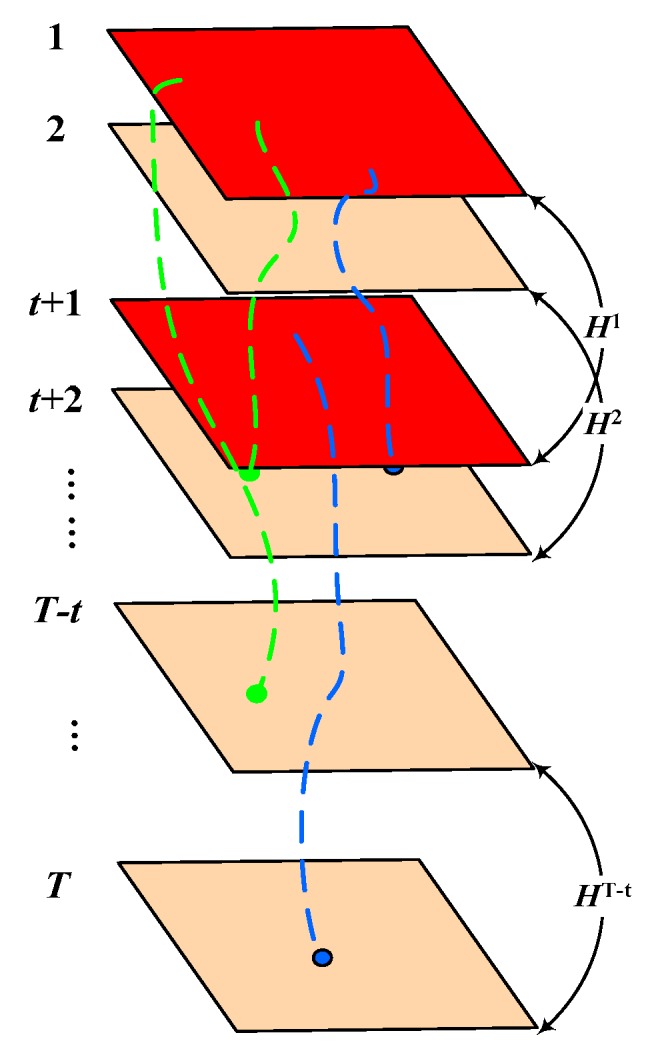
Illustration for the temporally partition of the input video.

As illustrated in Figure 2, two frames with interval t are regarded as adjacent frames. That is, for a video sequence with T frames, the corresponding multi-frame homography matrices are $H^{multi}=\left\{H^{k},k=1,\ldots ,T-t\right\}$. To calculate the homography matrix $H^{k}$ of each window, we applied the Random Sample Consensus (RANSAC) framework [37] to compute the best estimate of corresponding points that satisfy the homography model (inlier points). To improve the accuracy of homography matrix estimation, we used all the inlier points to re-estimate the homography constraint matrix after performing the RANSAC calculation. The matrix was then used to obtain a new set of inlier points. This method was iterated several times until the number of inlier points remains the same.

Obviously, the framework in relation to above homography matrix estimation easily demonstrations the ability to classify the long-term trajectories on each window by measuring the projection error. However, because of the irregularity of the object movement, the motion amplitude of the object may be small in some windows (such as the object is keeping still for a short period of time). If we perform the trajectories classification on each window, respectively, and merge the classification of successive windows to yield the final aggregate result, the classification accuracy will be low. Thus, in order to make use of the full motion cues of a certain trajectory in its life cycle, we propose a cumulative acknowledgment strategy to distinguish the properties of each trajectory. More precisely, we assumed that the first and last frame of the trajectory $\Lambda_{i}$ are $t_{i}$, and $T_{i}$, respectively. Then, based on our temporal partition illustrated in Figure 2, the corresponding homography matrix set of trajectory $\Lambda_{i}$ is:

(3) $$H_{i}^{\mathrm{multi}}=\left\{H^{k},k=t_{i},\ldots ,T_{i}-t\right\}$$

We define the average projection error of trajectory $\Lambda_{i}$ as:

(4) $$\varepsilon_{i}=\frac{1}{T_{i}-t-t_{i}+1}\sum_{k=t_{i}}^{T_{i}-t}\left\|x_{i}^{k+t}-H^{k}x_{i}^{k}\right\|$$

In general, if $\Lambda_{i}$ is the background trajectory, the corresponding average projection error $\varepsilon_{i}$ should be small. Conversely, if $\Lambda_{i}$ is the trajectory of moving object, $\varepsilon_{i}$ should be larger. So the trajectories can be well distinguished by the value of $\varepsilon_{i}$. In an ideal case, the trajectory can generally be classified by setting an appropriate threshold. However, there are often errors in the actual trajectory extraction. For example, some trajectory points drift often occurs in the boundary of the background and the moving object, which will result in some erroneous trajectories. According to our observation, the average projection error of these erroneous trajectories is often between the background trajectories and the moving object trajectories. Therefore, a double threshold method is proposed to remove the effect of these erroneous trajectories, as shown in the Equation (5):

(5) $$\Lambda_{i}=\{\begin{array}{cc} \mathrm{Background trajectory} & if\;\varepsilon_{i}\leq \tau_{L}\\ \mathrm{Foreground trajectory} & if\;\varepsilon_{i}\geq \tau_{H}\\ \mathrm{Noise trajectory} & \mathrm{otherwise} \end{array}$$

To further improve the trajectory classification, we use motion boundary to refine the spatial accuracy. Firstly, we compute the optical flow vector between pairs of subsequent frames. Let $f_{t}\left(i,j\right)$ be the optical flow vector at image position (i,j) of the t-th frame, $\left\|\vec{\nabla f_{t}\left(i,j\right)}\right\|$ be the corresponding gradient magnitude of optical flow. Then, we can define a strength ratio of motion boundary $b_{t}\left(i,j\right)$:

(6) $$b_{t}\left(i,j\right)=1-\exp\left(-\lambda \left\|\vec{\nabla f_{t}\left(i,j\right)}\right\|\right)$$

where λ is a parameter controlling $b_{t}\left(i,j\right)\in \left[0,1\right]$. The image points whose $b_{t}\left(i,j\right)$ is close to 1 (we set 0.8 in our experiments) are regarded as the physical object boundaries ${\hat{b}}_{t}$. Finally, the corresponding point of trajectory $\Lambda_{i}$ on the t-th frame is assigned the foreground/background label by the point in polygon problem.

Step1) Shoot 8 rays spaced by 45 degrees for the image point of trajectory $\Lambda_{i}$ on the t-th frame.

Step2) Calculate the intersection number of each ray and the estimated object boundaries ${\hat{b}}_{t}$. For each ray, if its intersection number is odd (or even), it can cast a vote of foreground (or background) label.

Step3) Assign foreground/background labels to image point of trajectory $\Lambda_{i}$ on the t-th frame by the majority rule.

## 3. Foreground Background Labeling Based on Markov Random Fields Model

**Figure 3:**
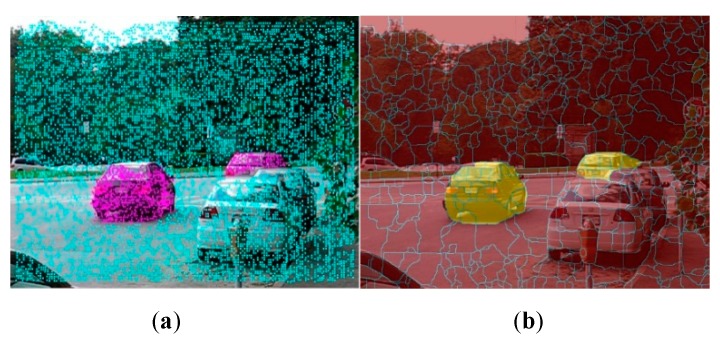
Example of initial trajectory classification and final pixel labeling. The selected image is from cars 2 video sequence of Hopkins 155 [40] dataset. (**a**) Example of initial trajectory classification. (**b**) Example of final pixel level labeling.

Motion information alone is often insufficient for acquiring the pixel level segmentation, since the tracked trajectories tend to be sparse relative to the image pixels. Indeed, only about 3% to 6% of image pixels are labeled as background or foreground by the proposed trajectory classification method. This is illustrated in Figure 3a, where the foreground and background trajectories are shown by the purple, and blue colors, respectively. To obtain a dense video segmentation, we propose an extension of graph cut [38,39] with per frame superpixel as node and convert trajectories classification to superpixels labeling. The superpixel can be defined as a group of pixels that share common characteristics (like pixel color). Using superpixels to deal with the dense video segmentation problem can ensure the pixels with similar color assign the same label while pixels with difference color assign opposite labels. Thus, it is more suitable for a basic unit of moving object segmentation problem, compared to the pixel-based method. Figure 3b shows the final segmentation result, where the boundary between the superpixels is expressed in blue line, and the background regions are darkened while the foreground ones are highlighted by the yellow color.

Specifically, we used SLIC algorithm [41,42] to oversegment each frame to obtain a set of superpixels. Let ${ℜ}_{t}$ denotes the superpixel set of the t-th frame. Each superpixel $r_{t}^{i}\in {ℜ}_{t}$ can correspond to a label $l_{t}^{i}\in \left\{0,1\right\}$, where 0 denotes the background and 1 denotes the foreground. Thus, we formulated the moving object segmentation as superpixel labeling problem by construct a spatial temporal MRF model with the superpixel set as node. And we designed an energy function to estimate the labeling,

(7) $$E\left(L\right)=\sum_{t,i}\sum_{c=0}^{1}A_{t}^{c}\left(r_{t}^{i}\right)+\sum_{t,i}\sum_{r_{t}^{j}\in \psi \left(r_{t}^{i}\right)}S_{t}^{i,j}\left(r_{t}^{i},r_{t}^{j}\right)\;+\sum_{t,i}\sum_{r_{t+1}^{j}\in \zeta \left(r_{t}^{i}\right)}T_{t}^{i,j}\left(r_{t}^{i},r_{t+1}^{j}\right)$$

where $L={\left\{l_{t}^{i}\right\}}_{t,i}$ denotes the output labeling of all the superpixels, $\psi \left(r_{t}^{i}\right)$ and $\zeta \left(r_{t}^{i}\right)$ denote the spatial and temporal neighborhood set of the superpixel $r_{t}^{i}$, $A_{t}^{c}\left(\cdot \right)$, $S_{t}^{i,j}\left(\cdot \right)$ and $T_{t}^{i,j}\left(\cdot \right)$ denote the unary potential, spatial smoothness potential, and temporal smoothness potential, respectively.

$A_{t}^{c}\left(\cdot \right)$ reflects the similarity that a superpixel is background or foreground according to the trajectory classification result obtained in the previous section. To get a better segmentation result, we design a dynamic appearance model of the foreground and background. Firstly, we calculate a coefficient $p_{t}^{i}$ according to the normalized histogram of foreground trajectory labels that intersect the interior of superpixel $r_{t}^{i}$. The larger the coefficient $p_{t}^{i}$, the more likely superpixel $r_{t}^{i}$ belongs to the foreground object, and vice versa. Thus, we can compare the coefficient $p_{t}^{i}$ with two thresholds and classify the superpixels into foreground and background preliminarily.

(8) $$r_{t}^{i}=\{\begin{array}{cc} \mathrm{foreground}\;\mathrm{superpixels} & if\;p_{t}^{i}\geq T_{1}\\ \mathrm{background}\;\mathrm{superpixels} & if\;p_{t}^{i}\leq T_{2} \end{array}$$

Then, we used a low-dimensional vector consisting of the centroid coordinate and average RGB color, to represent each superpixel. At each frame, t, we estimated the dynamic appearance model, including two Gaussian Mixture Models ($g_{t}^{1}$ denotes the foreground model and $g_{t}^{0}$ denote the background model) based on the initial superpixels classification results. Considering that the larger the coefficient $p_{t}^{i}$, the more devoting to the foreground appearance model $g_{t}^{1}$, we incorporated a weight to quantity this contribution. More precisely, the weight of each foreground superpixel is expressed as:

(9) $$\lambda_{t}^{i}=\frac{p_{t}^{i}}{\sum_{i}p_{t}^{i}}$$

While the estimation of the background appearance model $g_{t}^{0}$ is analogous, with the weight $\lambda_{t}^{i}$ replaced by:

(10) $$\lambda_{t}^{i}=\frac{p_{t}^{i}}{\sum_{i}\left(1-p_{t}^{i}\right)}$$

Finally, after estimating the foreground and background appearance models, the unary potential $A_{t}^{c}\left(r_{t}^{i}\right)$ is the log probability of superpixel $r_{t}^{i}$ with label $l_{t}^{i}$ under the appropriate model (i.e., the foreground model $g_{t}^{1}$ if $l_{t}^{i}=1$ and vice versa).

(11) $$A_{t}^{c}\left(r_{t}^{i}\right)=-\log\left(g_{t}^{c}\left(r_{t}^{i}\right)\right)\cdot \delta \left(l_{t}^{i},c\right)$$

where δ(·) is the Kronecker delta function. The above equation shows that if a superpixel is given a label, which is more consistent with its appearance model, its unary potential will be small, so that the whole energy function are ensured to be minimum.

The pairwise potentials are used to encode the continuity of the adjacent superpixels, and can be divided into two types: spatial smoothness potential and temporal smoothness potential. For the spatial smoothness, we consider two superpixels to be spatially connected if they are adjacent and in the same frame. Then, we define the pairwise cost as,

(12) $$S_{t}^{i,j}\left(r_{t}^{i},r_{t}^{j}\right)=\left(1-\delta \left(l_{t}^{i},l_{t}^{j}\right)\right)\cdot \;\frac{1}{\left\|c_{t}^{i}-c_{t}^{j}\right\|}\cdot \frac{1}{e^{\left\|h_{t}^{i}-h_{t}^{j}\right\|}}$$

where $r_{t}^{j}\in \psi \left(r_{t}^{i}\right)$, $\left\|c_{t}^{i}-c_{t}^{j}\right\|$ is the Euclidean distance between the centroid coordinate of two superpixels and $e^{\left\|h_{t}^{i}-h_{t}^{j}\right\|}$ is the difference between the average color of two superpixels. For the temporal smoothness, we consider two superpixels to be temporally connected if they are in subsequent frames and there is at least one pixel after optical flow compensation [43,44]. We define $r_{t+1}^{j}\in \zeta \left(r_{t}^{i}\right)$ as the temporal neighborhood of superpixel $r_{t}^{i}$, and we assume $Q\left(r_{t}^{i},r_{t+1}^{j}\right)$ to be the overlap area of $r_{t}^{i}$ moving into $r_{t+1}^{j}$ after the optical flow compensation. Then, we define the pairwise cost as:

(13) $$T_{t}^{i,j}\left(r_{t}^{i},r_{t+1}^{j}\right)=\left(1-\delta \left(l_{t}^{i},l_{t+1}^{j}\right)\right)\cdot \;Q\left(r_{t}^{i},r_{t+1}^{j}\right)\cdot \frac{1}{e^{\left\|h_{t}^{i}-h_{t+1}^{j}\right\|}}$$

After establishing the potential function of each superpixel, we used a graph cut algorithm to solve the energy minimization problem, and get the optimal classification result of each superpixel. In summary, all steps of the proposed video object segmentation algorithm are summarized in Algorithm 1.

**Algorithm 1** Video segmentation algorithm based on multi-frame homography constraints1: **Input:** video sequence 2: **Initialize:** frames number *T*; temporal window interval *t*; trajectory classification thresholdand $\tau_{L}$ and $\tau_{H}$; initial superpixel classification parameters *T*_1_ and *T*_2_3: **Trajectory classification based on multi-frame homography model** a: Calculate the long term trajectory $\Lambda =\left\{\Lambda_{i},i=1,\ldots ,n\right\}$ of input video b: Estimate the homography matrix set $H^{multi}=\left\{H^{k},k=1,\ldots ,T-t\right\}$ c: for i=1,…,n do  Use Equation (3) to select the corresponding homography matrix set $H_{i}^{multi}$ of trajectory $\Lambda_{i}$  Use Equation (4) to estimate the average projection error $\varepsilon_{i}$ of the trajectory $\Lambda_{i}$  end for d: Use Equation (5) to classify the motion trajectory.  e: Use motion boundary to refine the spatial accuracy of trajectory classification.4: **Pixel labeling based on Markov Random Fields model**
 f: Oversegment the input video to get the superpixel set $\sum r_{t}^{i}$
 g: for *t* = 1:*T* do   Use Equation (8)–(11) to calculate the unary potential $A_{t}^{c}\left(r_{t}^{i}\right)$ of superpixel $r_{t}^{i}$
  Use Equation (12) and (13) to calculate the pairwise potential $S_{t}^{i,j}\left(r_{t}^{i},r_{t}^{j}\right)$ and $T_{t}^{i,j}\left(r_{t}^{i},r_{t+1}^{j}\right)$ of superpixel $r_{t}^{i}$
  end for h: Use graph cut algorithm to solve the energy function minimization problem5: **Output:** Pixel level object segmentation result for each frame of input video

## 4. Experiments

Several publicly released video sequences are selected as experimental data in this study. These videos are from the Hopkins dataset [40] (cars1–7, people1–2), and from [34] (vcar, vperson, vhand), which have been used by recent quantitative papers on this topic [18,19,20,21,22,23,24,25,26,27,28,29,30,31,32]. We additionally include a challenging sequence that reflects a real surveillance scene acquired by the PTZ camera [45] (backyard) and three standard videos captured by the stationary camera (highway, office, and pets2006) [46]. The selected videos, include rapidly camera motion, non-rigid deformations, multiple moving objects, and arbitrary changes in the scene content.

In order to obtain real the background and foreground object regions, we manually generated ground truth data by extracting the moving objects in every five frames. In our experiments, we set *t* = 10, which means each overlapped window contains ten frames. The trajectory classification threshold is kept fixed at $\tau_{L}=0.01$, $\tau_{H}=0.05$ in all experiments. The initial classification parameters of superpixel are set as T_1_ = 0.2, T_2_ = 0.01. Our experiments are conducted on a PC with dual-core Intel i7 Ivy bridge 2.50 GHZ CPU. The proposed method is implemented using a combination of C++ and MATLAB code.

### 4.1. Experimental Results on Trajectory Classification

**Figure 4:**
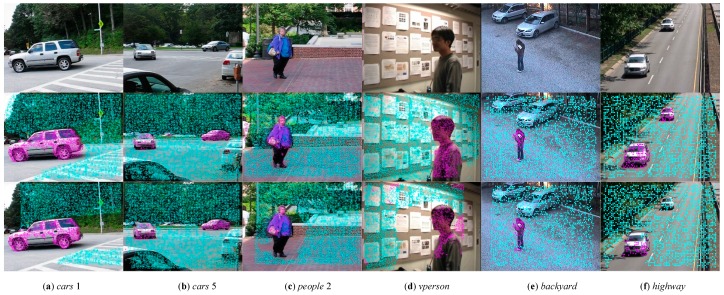
Trajectory classification results on the *cars* 1 (column 1), *cars* 5 (column 2), *people* 2 (column 3), *vperson* (column 4), *backyard* (column 5), and *highway* (column 6) sequences using our method (row 2) and Sheikh et al. [22] (row 3). For visualization purposes, the foreground and background trajectories are shown by the purple, and blue colors, respectively.

We firstly present the experimental results on trajectory classification with comparison to low rank based method [22]. When testing our method, the same trajectories were used for both algorithms. The qualitative results on the trajectory level classification are shown in Figure 4. Figure 4a gives an example, where multiple objects are separated and then occluded. It can be seen that the proposed method outperforms the low rank-based method on both trajectory distribution and classification accuracy. Figure 4b presents an experimental result, where multiple objects have large scale differences. The low rank-based method employs affine camera model, which is difficult to describe the depth and scale changes. This limitation results in labeling the long-range small car on the middle of the image, as part of background. However, our algorithm detects it correctly as foreground region. Figure 4c is another example that explains our proposed multiple overlapped homography constraint well. In this case, the white clothed pedestrian leaves the camera’s field of view in about twentieth frame. Due to the entire usage of tracked trajectories, our method can make trajectory points uniformly distributed in the image plane, which will greatly benefit the final pixel level labeling. Figure 4d presents an experimental result of an indoor surveillance video. The selected sequence is challenging because it includes no-rigid deformations and scale changes. The moving person has similar color to the background. As we can see from the experimental result, our method also performs well in a challenging scene. This demonstrates the ability of our multi-frame homography background representation. Figure 4e gives a difficult example, where the video sequence is clipped from a PTZ camera on real surveillance scene. On the long sequence, the moving pedestrian remains still for a period of time. Methods in [22] face difficulty in this situation; the results have an obvious classification error at the leg of the pedestrian. Benefit from the long term motion analysis and cumulative acknowledgment strategy, our method can still classify the background trajectories and foreground ones successfully. Figure 4f is a highway surveillance video taken by a stationary camera. The rank of background trajectory matrix is two, which does not satisfy the fixed rank constraint (i.e., three) of the low-rank constraint algorithm [22]. Thus, when the algorithm is used to separate trajectories, several foreground trajectories are misclassified as background ones. By contrast, the proposed algorithm models the background motion of adjacent frames as homography constraint, which can still adapt to stationary cameras. So it can accurately separate background and foreground trajectories.

We also present quantitative performance evaluation of our algorithm, compared to low rank-based method [22] in trajectory classification. For quantitative comparison, we considered the percentage of correct classification (PCC) and trajectory distribution (Density) in our experiments, which are defined as follows [47]:

(14) $$\{\begin{array}{c} \mathrm{PCC}=\frac{\mathrm{TP}+\mathrm{TN}}{\mathrm{TP}+\mathrm{FP}+\mathrm{TN}+\mathrm{FN}}\\ \mathrm{Density}=\frac{\mathrm{TP}+\mathrm{FP}+\mathrm{TN}+\mathrm{FN}}{\mathrm{Width}\times \mathrm{Height}} \end{array}$$

The TP, FP, TN, and FN denote the number of true-positive, false-positive, true-negative, and false-negative trajectories, respectively. The Width and Height represent the size of tested image. Using above metrics, we get the quantitative results on the trajectory level separation as shown in Table 1. In summary, our method obviously outperformed the low rank based method [22] in PCC evaluation. This is because we divided the whole video into multiple overlapped windows, whereby the background motion of each window could be represented by the homography model. Thus, all the trajectories could be distinguished by the multi-frame homography constraint, based on the cumulative acknowledgment strategy, which make motion information accumulated in the life cycle of long term trajectory fully utilize. Furthermore, we observed that [22] has no trajectories around the moving objects and image boundaries since the incomplete trajectories are discarded in their method. Thus, our method gets an obvious higher density of trajectories than [22]. Both, the higher classification accuracy and trajectory densities is beneficial for subsequent pixel level labeling.

### 4.2. Experimental Results on Pixel Level Labeling

**Figure 5:**
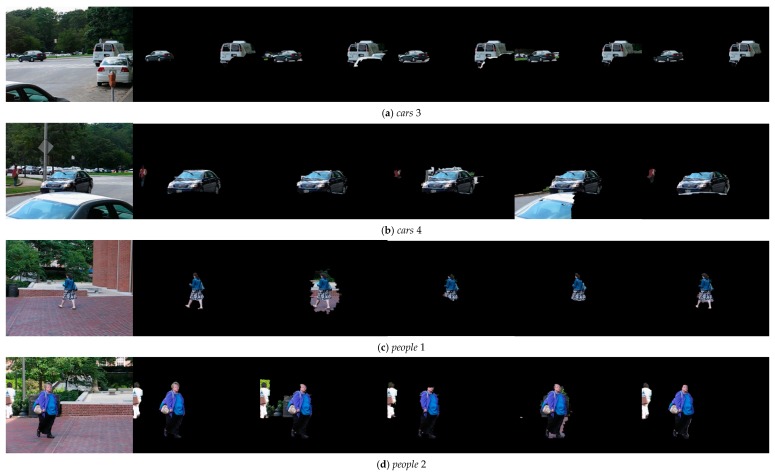

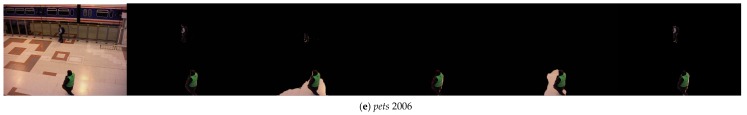
Experimental results of final pixel level labeling on the *cars* 3 (row 1), *cars* 4 (row 2), *people* 1 (row 3), *people* 2 (row 4), and *pets* 2006 (row 5) sequences. (Column 1) Input image. (Column 2) Ground truth. (Column 3) Sheikh et al. [22]. (Column 4) Zhu et al. [26]. (Column 5) Chiranjoy et al. [31]. (Column 6) Our algorithm.

We also evaluated the performance of our method with several start-of-the-arts method at the pixel level labeling. The compared methods are trajectory-based methods [22,26] and optical flow-based method [31]. The results on the pixel-level labeling are shown in Figure 5. Obviously, our method outperforms the competitive methods. As presented in cars 4 and people 2 sequences, we observed that [22] is prone to error around the moving object and image boundaries, since it has no trajectory at these regions. Furthermore, this method lacks the appearance models and spatial temporal constraints at pixel-level labeling, which will cause inconsistent segmentations of moving objects. The method presented in [26] relies on block-based motion and appearance model estimation and propagation. While, we found that their method is not stable and sensitive to parameter selection through our pixel level labeling experiments. We also observed that [31] has lower segmentation precision when the object is small and moving non-rigidly, such as people 1 sequences. The reason is that this algorithm relies on bottom-up saliency detection which is difficult when non-rigid deformations happen in the moving object.

In Table 2, we present quantitative comparison results, using well-known F-measure metric [48,49], which are computed, based on the labels generated by the algorithms and manually annotated ground truths. It should be point that we report the F-measure metric of [26] based on the results reported in the original paper. In addition, we also add three additional algorithms for quantitative comparison, where their results have been released on several papers [26,27,28,35]. From the results shown in Table 2, we can confirm that our approach produce almost the highest F-measure scores compared other six methods. Moreover, our F-measure scores are more than 85% in all test videos. The reason is because we, not only obtained a higher trajectory classification accuracy in advance, but also introduced appearance model and spatial temporal smoothness in pixel labeling stage. This greatly refines the initial segmentation compared to the use of motion information only.

## 5. Conclusions

We present a novel and modular object segmentation algorithm for both, a stationary camera and a moving camera. In order to take full use of motion information and cover videos sufficiently, we divided the input sequences into several overlapping temporal windows. Then, multi-frame background model was built, based on homography constraint of each window, and cumulative acknowledgment mechanism is introduced for the trajectory classification. We incorporated a trajectory classification, dynamic appearance, spatial temporal information for the final pixel labeling, which can automatically refine the spatial accuracy of the trajectory-based segmentation and to also segment the objects in frames.

In order to make an accurate evaluation, we compared the proposed method with the state-of-the-art approaches on multiple challenging videos. The comparisons are evaluated from two aspects: One is the performance of the trajectory classification and the other is the performance of the final pixel level labeling. Quantitative and qualitative experiments demonstrate that the proposed method achieves promising performances from these two aspects, respectively.

Similar to all previous methods, our algorithm works in offline mode. Thus, it is not suitable for real-time moving object segmentation. In the future, we plan to develop an online version of a proposed algorithm that can work incrementally, e.g., the multi-frame homography constraint, extracted from the beginning frames can be updated online when the new frames arrive.

## Figures and Tables

**Table 1:** Quantitative evaluation of motion trajectory classification.

Video Sequence	Sheikh et al. [22]		Our Method	
	PCC/%	Density	PCC/%	Density
*cars1*	97.51	0.0432	98.89	0.0490
*cars2*	98.23	0.0483	99.56	0.0502
*cars3*	98.66	0.0480	99.48	0.0521
*cars4*	97.49	0.0489	99.40	0.0537
*cars5*	97.90	0.0511	99.29	0.0528
*cars6*	99.74	0.0449	99.88	0.0496
*cars7*	98.93	0.0457	99.52	0.0502
*people1*	99.45	0.0469	99.93	0.0513
*people2*	98.23	0.0436	99.94	0.0498
*vcar*	98.64	0.0479	99.65	0.0510
*vperson*	98.92	0.0387	99.51	0.0477
*vhand*	97.48	0.0430	99.10	0.0531
*backyard*	99.10	0.0384	99.81	0.0464
*highway*	97.89	0.0412	99.45	0.0531
*office*	96.38	0.0406	99.11	0.0479
*pets2006*	97.16	0.0387	98.94	0.0566

**Table 2:** Quantitative evaluation of pixel level labeling.

Video Sequence	Sheikh et al. [22]	Zhu et al. [26]	Chiranjoy et al. [31]	Lim et al. [27]	Kwak et al. [28]	Zamalieva et al. [35]	Our Method
*cars1*	0.823	0.920	0.847	0.871	0.803	0.822	0.936
*cars2*	0.839	0.902	0.778	0.822	0.685	0.789	0.892
*cars3*	0.834	0.932	0.724	0.729	0.792	0.882	0.933
*cars4*	0.822	0.916	0.646	0.882	0.666	0.895	0.889
*cars5*	0.821	0.866	0.661	0.817	0.746	0.874	0.873
*cars6*	0.893	0.922	0.827	0.814	0.733	0.903	0.931
*cars7*	0.846	0.912	0.751	0.909	0.691	0.869	0.928
*people1*	0.829	0.814	0.453	0.812	0.802	0.866	0.890
*people2*	0.904	0.943	0.642	0.847	0.814	0.916	0.941
*vcar*	0.837	0.879	0.618	x	x	x	0.896
*vperson*	0.799	0.865	0.709	x	x	x	0.928
*vhand*	0.671	0.846	0.611	x	x	x	0.876
*backyard*	0.829	0.772	0.715	x	x	x	0.884
*highway*	0.537	0.684	0.464	x	x	x	0.879
*office*	0.686	0.702	0.712	x	x	x	0.861
*pets2006*	0.684	0.769	0.632	x	x	x	0.892

## Supplementary Files

Supplementary File 1
